# Gastrointestinal Infection Before Immune Checkpoint Inhibition Hinders Treatment Efficacy and Increases the Risk of Colitis

**DOI:** 10.1002/cam4.71123

**Published:** 2025-08-10

**Authors:** Malek Shatila, Kian Abdul‐Baki, Andres Urias Rivera, Kei Takigawa, Irene Jeong‐Ah Lee, Andrew Sullivan, Tanvi Gupta, Linfeng Lu, Raakhi Menon, Ayesha Khan, Hamza Salim, Elliot Baerman, Carolina Colli Cruz, Cristina Natha, Varun Vemulapalli, Garrett Coleman, Krishnavathana Varatharajalu, Christopher Fan, Pablo Okhuysen, Anthony J. Olszanski, Yan Zhou, Hao Chi Zhang, Mehnaz Shafi, Yinghong Wang

**Affiliations:** ^1^ Department of Gastroenterology, Hepatology, and Nutrition The University of Texas MD Anderson Cancer Center Houston Texas USA; ^2^ Department of Internal Medicine The University of Texas Medical Branch Galveston Texas USA; ^3^ Department of Internal Medicine Baylor College of Medicine Houston Texas USA; ^4^ Department of Internal Medicine The University of Texas Health Sciences Center Houston Texas USA; ^5^ Department of Internal Medicine Memorial Hermann Hospital Houston Texas USA; ^6^ Department of Gastroenterology Houston Methodist Hospital Houston Texas USA; ^7^ Department of Infectious Diseases, Infection Control and Employee Health The University of Texas MD Anderson Cancer Center Houston Texas USA; ^8^ Department of Medical Oncology Fox Chase Cancer Center Philadelphia Pennsylvania USA; ^9^ Department of Hospital Medicine The University of Texas MD Anderson Cancer Center Houston Texas USA

**Keywords:** gastrointestinal infections, gut microbiome, immune checkpoint inhibitors, immune‐mediated colitis, intestinal dysbiosis, risk factors, selective immunosuppressive therapy

## Abstract

**Background:**

Gastrointestinal (GI) infections, which often result in or stem from intestinal dysbiosis, can affect the efficacy of immune checkpoint inhibitors (ICIs) and increase the risk of adverse effects, such as colitis. In this study, we explored the impact of GI infections before initiation of ICI therapy on the incidence and severity of immune‐mediated colitis (IMC) and survival.

**Methods:**

A single‐center, retrospective review including all patients who received ICIs from January 2010 to February 2024 and subsequently developed IMC. Patients were screened for IMC and prior GI infections based on symptoms and stool tests. Patients' demographic, IMC, and GI infection–related clinical data were collected.

**Results:**

Thirty‐four of the 1132 patients (3.0%) included in the analysis had GI infections before ICI therapy. GI infections were most commonly caused by *Clostridioides difficile* and most often treated with oral antibiotics (interquartile range [IQR], 7–14 days). The incidence of IMC was higher in patients with prior GI infections compared to patients without prior infections (8.7% vs. 5.1%, *p* = 0.002). IMC symptoms, severity, and outcomes were similar in both groups (*p* > 0.05). In multivariate Cox proportional survival analysis, prior GI infection was independently associated with an increased risk of mortality (odds ratio, 1.6 [95% CI, 1.4–1.8]; *p* < 0.001) for patients who received ICIs.

**Conclusions:**

Our study is the first to explore the impact of GI infection before ICI therapy on IMC risk and survival. We found that prior GI infection was associated with an increased incidence of IMC and an increased risk of mortality in patients receiving ICIs.

**Abbreviations**
FMT: fecal microbiota transplantation
GI: gastrointestinal
ICI: immune checkpoint inhibitor
IMC: immune‐mediated colitis
IRAE(s): immune‐related adverse event(s)

## 1 Introduction

In recent years, the gut microbiome has received increasing attention for its role in cancer treatment responsiveness and the development of treatment‐related toxicities, in part because of its' essential role in immune regulation through various mechanisms [1]. Gastrointestinal (GI) infections can result from and contribute to intestinal dysbiosis, a state of microbial imbalance characterized by a reduction in the diversity of the microbial taxa, allowing pathogenic bacteria to outcompete commensal organisms, leading to infection [2]. Gut dysbiosis has been associated with reduced efficacy of immune checkpoint inhibitors (ICIs) and an increased risk of immune‐related adverse events (IRAEs), including immune‐mediated colitis (IMC), which is the most common IRAE requiring the cessation of immune checkpoint inhibitor (ICI) therapy, thereby compromising cancer treatment outcomes [3, 4, 5].

Intestinal dysbiosis has been associated with increased rates of IMC, increased need for hospitalization, and increased use of immunosuppressive therapy to manage IMC symptoms, particularly in patients who have received antibiotics before ICI therapy [3]. Patients who receive antibiotics before ICI therapy have worse overall survival outcomes, delayed cancer response, and lower overall cancer response rates compared to patients who do not [6, 7]. Furthermore, concomitant GI infections during ICI administration are associated with more severe IMC symptoms [8].

The baseline microbiota composition may predict the risk of IMC, highlighting the potential for gut microbiome profiling in identifying patients at risk [9]. Fecal microbiota transplantation (FMT) is known to provide rapid and substantial symptom relief in patients with IMC regardless of concurrent *Clostridioides difficile* infection [10, 11]. Notably, patients undergoing ICI therapy are also susceptible to opportunistic infections, such as cytomegalovirus (CMV), which can mimic IMC symptoms, complicating the diagnosis of IMC [12]. Current American Society of Clinical Oncology guidelines recommend ruling out infectious etiologies through stool pathogen testing before initiation of antibiotic therapy unless otherwise clinically indicated in patients on ICIs who develop new‐onset diarrhea.

Although researchers have previously explored the relationship among intestinal dysbiosis, ICI efficacy, and IMC severity, to our knowledge, no studies have specifically examined the impact of GI infections occurring before immunotherapy on the incidence and severity of GI IRAEs after ICI therapy. To address this gap, we explored how preexisting GI infections influence the risk of developing IMC and overall survival in patients undergoing ICI treatment.

## 2 Methods

### 2.1 Patient Selection

We conducted a retrospective, single‐center study at the University of Texas MD Anderson Cancer Center. We included patients who received ICIs between January 1, 2010 and February 31, 2024, and who subsequently developed IMC. Patients were divided into two groups: those who had GI infections within 3 months before the initiation of ICI therapy and those who did not. We excluded patients with inadequate documentation of any GI IRAEs in their medical records and those with nonimmune‐mediated etiologies of esophagitis, gastroenteritis, or colitis.

### 2.2 Identification of GI IRAEs and GI Infections

To identify IMC, we retrieved stool infectious (*Clostridioides difficile* testing, GI multiplex pathogen panel) and inflammatory (fecal lactoferrin and calprotectin) workup and lower endoscopy data on all patients treated with ICIs. These cases were then independently screened to identify confirmed or strongly suspected IMC cases based on physicians' clinical judgment, as well as objective biomarker (negative infectious workup, positive inflammatory markers) or biopsy results. Patients were excluded if other etiologies for their GI inflammation, such as ischemic, infectious, tumor‐related, or autoimmune causes, were identified.

To identify patients with GI infections prior to immunotherapy initiation, we retrieved data on all stool infectious workup done within 3 months before ICI initiation. The rationale behind choosing a 3‐month infectious time window is due to reports in the literature that suggest postinfectious intestinal dysbiosis can last for up to 14 weeks [13, 14, 15]. We then reviewed cases with stool infectious workup ordered and included all patients who had a pathogen identified or had documentation of an active infection. Patients with no such workup done were included in the “no infection” group.

### 2.3 Data Collection

We collected patient demographic information, such as age, sex, race, cancer type, ICI treatment details, vital status, and date of last follow‐up visit or death. We also collected data on clinical IRAE characteristics, such as symptoms, severity grading using the Common Terminology Criteria for Adverse Events (version 5), IMC treatment, and outcomes, such as IRAE resolution, recurrence, and hospitalization. Furthermore, we collected data on GI infections within 3 months before the initiation of ICI therapy and relevant clinical characteristics, such as presenting symptoms, implicated pathogens, stool studies, and the recurrence of infection after ICI treatment but prior to IMC diagnosis. Lastly, we collected data on antibiotic treatments related to GI infections, such as choice of antibiotic, its mechanism of action, its route of administration, and treatment duration.

### 2.4 Primary and Secondary Outcomes

The primary outcomes were the effect of GI infections before ICI therapy on the severity of lower GI IRAEs as measured by using the Common Terminology Criteria for Adverse Events, the need for steroids or selective immunosuppressive therapy (SIT), and IRAE resolution rates. Our secondary outcome was the impact of GI infections before ICI therapy on overall survival among patients receiving immunotherapy.

### 2.5 Statistical Methods

**FIGURE 1:**
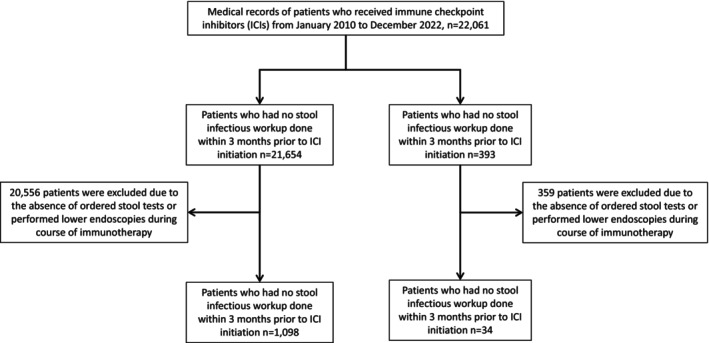
Patient selection flowchart.

Statistical analyses were conducted using SPSS software (version 26.0). Continuous variables were described using median values and interquartile ranges (IQRs), and categorical variables were described using frequencies and percentages. Differences between categorical variables were compared using the Fisher exact test; Bonferroni correction was applied for multiple comparisons when categorical variables had more than two levels. The Mann–Whitney *U* test was used to compare medians for continuous variables. Multivariate Cox proportional hazards survival analysis was used to compare overall survival from the date of first ICI dose in the two groups; cases that were lost to follow‐up were censored in the final analysis. Sample size calculations were performed using the population proportions found in Figure 1, with a designated alpha of 0.05 and power of 0.95. A total sample of at least 28 patients with GI infection would be needed to achieve adequate study power. *p* values < 0.05 were considered significant.

## 3 Results

### 3.1 Demographics

Overall, we included 1132 patients with IMC in this study, 34 of whom had a GI infection before ICI initiation and 1098 of whom did not (Figure 1). The cohort had a median age of 65 years (IQR, 55–72 years) and was predominantly male (58.8%) and White (89.7%). Patients received immunotherapy for a median of 5.5 months (IQR, 1.6–15.3 months). The most commonly used ICIs were anti‐PD1/L1 agents (49.1%), followed by combination therapy (34.2%) and anti‐CTLA‐4 agents (16.6%). Melanoma was the most common cancer type (34.9%), followed by genitourinary (22.7%) and lung cancers (12.8%). The majority (87.8%) of patients had an Eastern Cooperative Oncology Group performance score of 0 or 1. About half of the patients died by the end of the study, with a median length of follow‐up of 1.5 years. Patient demographic characteristics are presented in Table 1.

**TABLE 1:** Patient demographic characteristics (*n* = 1132).

Characteristic	*N* (%)
Median age at the time of immunotherapy, year (IQR)	65 (55–72)
Male sex	666 (58.8)
Race–White	1016 (89.7)
Type of ICI	
Anti‐PD‐1/PD‐L1	556 (49.1)
Anti‐CTLA‐4	188 (16.6)
Combination	388 (34.2)
Median duration of immunotherapy, month (IQR)	5.5 (1.6–15.3)
Cancer type	
Melanoma	400 (35.3)
Genitourinary	257 (22.7)
Lung	145 (12.8)
Gastrointestinal	94 (8.3)
Head and neck	55 (4.9)
Other	181 (15.9)
Cancer stage	
I	38 (3.4)
II	57 (5.0)
III	208 (18.4)
IV	790 (69.8)
ECOG performance status	
0	494 (43.6)
1	500 (44.2)
2–4	135 (11.9)
GI infection prior to ICI initiation^a^	34 (3.0)
All‐cause mortality	524 (46.2)
Length of follow‐up, median (IQR), year	1.5 (0.5–3.3)

Abbreviations: CTLA‐4, cytotoxic T‐lymphocyte antigen 4; ECOG, Eastern Clinical Oncology Group; GI, gastrointestinal; ICI, immune checkpoint inhibitor; IQR, interquartile range; PD‐1, programmed death 1; PD‐L, programmed death ligand‐1.

^a^ There were no significant differences in baseline characteristics between patients who had and did not have GI infection within 3 months prior to ICI initiation, including variables such as NSAID and PPI use that are not shown here.

### 3.2 Characteristics of Pre‐ICI GI Infection

As described above, a total of 34 patients developed a GI infection before the start of ICI treatment (Table 2). *C. difficile* was implicated in 66.7% of these infections, with *Escherichia coli* affecting 18.2% of patients. Diarrhea was the primary presenting symptom in 96.9% of cases, with abdominal pain occurring in 25.0% and hematochezia occurring in 9.4%. Most patients (86.2%) received oral antibiotics, with more patients receiving vancomycin/fidaxomicin‐based regimens than ciprofloxacin/metronidazole‐based regimens (72.4% vs. 27.6%, respectively). Additional information on GI infection characteristics is provided in Table 2.

**TABLE 2:** Clinical characteristics of gastrointestinal infections prior to ICI therapy (*n* = 34)^a^.

Characteristic	*N* (%)
Presenting symptoms	
Diarrhea	31 (96.9)
Abdominal pain	8 (25.0)
Blood or mucus in stool	3 (9.4)
Pathogen	
*C. difficile*	22 (66.7)
*E. coli*	6 (18.2)
Viral	2 (6.1)
Other	3 (9.1)
Antibiotics used	29 (87.9)
Type of antibiotic	
Ciprofloxacin/metronidazole‐based regimen	8 (27.6)
Vancomycin/fidaxomicin‐based regimen	21 (72.4)
Administration route	
Oral	25 (86.2)
Parenteral	4 (13.8)
Median duration of antibiotic administration, day (IQR)	10 (7–14)

Abbreviation: IQR, interquartile range.

^a^ Thirty‐four patients had confirmed gastrointestinal infection prior to ICI therapy. On chart review, data on some variables was missing. For instance, only 32 patients had symptom descriptions, 33 had information on what pathogen was involved, and 29 had information on the antibiotic regimen used, as well as the administration route.

### 3.3 IMC Clinical Characteristics in the Infectious and Noninfectious Groups

A comparison of IMC clinical characteristics of patients with and without prior GI infections is presented in Table 3. The incidence of colitis was significantly higher (*p* = 0.002) among patients with GI infection before ICI (8.7% [34/393]) compared to those without infection (5.1% [1098/21,736]). Patients with GI infections before ICI therapy were 1.8 times more likely to develop IMC (*p* = 0.002) (Table S1). IMC tended to occur earlier (*p* = 0.051) in patients with a GI infection before ICI (median, 1.8 months) than in those without such an infection (median, 3.3 months). We found no differences in terms of presentations, treatments, or outcomes between the two groups (*p* > 0.05) except for FMT, which was more common in the group with prior GI infection (14.7% vs. 4.9%; *p* = 0.029). The efficacy of FMT for colitis did not differ significantly between the groups, as indicated by no differences in rehospitalization rates, resumed ICI therapy rates, endoscopic remission, or symptom resolution and duration (Table S2).

**TABLE 3:** Clinical colitis features among patients who did and did not have gastrointestinal infections prior to ICI therapy initiation (*n* = 1132).

Feature	No. (%)		*p*
	No GI infection before ICI (*n* = 1098)^a^	GI infection before ICI (*n* = 34)^a^	
Diagnosis of IMC^b^	1098 (5.1)	34 (8.7)	0.002*
Median time from ICI administration to IMC, m (IQR)	3.3 (1.4–8.6)	1.8 (0.9–6.2)	0.051
Other irAE^c^	317 (29.5)	4 (13.8)	0.095
CTCAE diarrhea grade			1.000
1	208 (19.8)	6 (18.8)	
≥ 2	840 (80.2)	26 (81.3)	
CTCAE colitis grade			0.366
1	587 (56.3)	15 (46.9)	
≥ 2	456 (43.7)	17 (53.1)	
Presenting symptoms			
Diarrhea	1041 (98.2)	34 (100)	1.000
Abdominal pain	408 (38.5)	17 (53.1)	0.101
Fever	122 (11.5)	4 (12.5)	0.780
Blood or mucus in stool	145 (13.7)	4 (12.5)	1.000
Median baseline calprotectin levels, μg/g (IQR)^d^	239 (67–685)	310 (57–777)	0.934
Treatment			
Supportive	971 (90.2)	32 (94.1)	0.765
Steroids	807 (73.8)	23 (67.6)	0.432
SIT^e^	494 (45.2)	12 (35.3)	0.296
FMT	54 (4.9)	5 (14.7)^f^	0.029*
Median duration of steroid administration, day (IQR)	31 (21–56)	30 (18–51)	0.467
Median number of steroid tapers, (IQR)	1 (1–2)	2 (1–2)	0.211
Median number of SIT doses, median (IQR)	3 (2–4)	3 (2–5)	0.584
Outcomes			
Median duration of IMC symptoms, day (IQR)	30 (12–66)	21 (10–67)	0.666
Colitis clinical remission	898 (84.8)	28 (93.3)	0.297
Colitis recurrence	293 (27.1)	10 (30.3)	0.693
Hospitalization for colitis	659 (60.3)	19 (55.9)	0.598
Median length of hospitalization, day (IQR)	6 (4–9)	8 (5–13)	0.185
Multiple hospitalizations	262 (40.4)	8 (42.1)	1.000
ICI therapy discontinued	831 (77.7)	23 (69.7)	0.291
ICI therapy resumed^g^	295 (35.5)	9 (39.1)	0.826
All‐cause mortality	508 (46.6)	16 (48.5)	0.861
Median length of follow‐up, year (IQR)	1.6 (0.6–3.3)	0.8 (0.3–2.2)	0.043*

*Note:* *Significant at *p* < 0.05.

Abbreviations: CTCAE, Common Terminology Criteria for Adverse Events; FMT, fecal microbiota transplantation; GI, gastrointestinal; ICI, immune checkpoint inhibitor; IMC, immune‐mediated colitis; IQR, interquartile range; IRAEs, immune‐related adverse event; SIT, selective immunosuppressive therapy.

^a^ These numbers reflect the total number of patients with or without GI infection prior to ICI. Since cases may be missing for the variables that follow, the percentages provided in the subsequent rows are a percentage of the available cases for each variable, and not the total number of patients.

^b^ The percentages here reflect the fraction of all patients receiving immunotherapy without (*n* = 21,736) or with (*n* = 393) prior GI infection.

^c^ Other IRAEs include skin, kidney, liver, joint, upper gastrointestinal, heart, lung, thyroid, and pituitary toxicities.

^d^ The reference range for calprotectin levels is < 50.0 μg/g.

^e^ SITs included infliximab, vedolizumab, and ustekinumab.

^f^ Four (80%) of these patients had *C. difficile* infection prior to immunotherapy.

^g^ The denominator for these rows is based on the total number of patients who received ICIs.

### 3.4 IMC Endoscopic Features

Endoscopic features of colitis in the two study groups are compared in Table 4. Of all the included patients, approximately two‐thirds underwent endoscopic evaluation of their colitis. We observed no significant differences in endoscopic or histological features between the two groups (*p* > 0.05). Among patients without a prior GI infection, nonulcerative inflammation was frequently seen upon gross examination (30.9% of patients), and acute active inflammation was the most common histological feature (51.2% of patients). Most patients (70%) had resolution of colitis based on endoscopic findings at the last follow‐up visit.

**TABLE 4:** Endoscopic colitis features among those who did and did not have gastrointestinal infections prior to ICI therapy initiation (*n* = 730).

Feature	*N* (%)		*p*
	No prior GI infection (*n* = 709)	Prior GI infection (*n* = 21)	
Endoscopy	709 (66.5)	21 (61.8)	0.583
Endoscopic findings^a^			
Normal	259 (38.1)	11 (52.4)	0.112
Nonulcerative inflammation	268 (39.5)	9 (42.9)	
Ulcerative inflammation	152 (22.4)	1 (4.8)	
Histopathological findings^a^			
Normal	110 (16.4)	3 (15.8)	1.000
Acute active inflammation	343 (51.2)	8 (42.1)	0.491
Chronic inflammation	115 (17.2)	2 (10.5)	0.755
Microscopic colitis	60 (9.0)	3 (15.8)	0.405
Colitis remission based on endoscopy at last follow‐up	145 (77.5)	5 (71.4)	0.658

Abbreviations: GI, gastrointestinal; ICI, immune checkpoint inhibitor.

^a^ Some patients who underwent endoscopy had missing data on the endoscopic and histological findings. Denominators for these categories may be different from those reported in the column header. For endoscopic findings, 679 patients had no prior GI infection compared to 21 with prior infection; for histological findings, 670 patients had no prior GI infection compared to 19 with prior infection.

**TABLE 5:** Multivariate Cox hazard analysis of the association between survival and gastrointestinal infection before immunotherapy.

Characteristic	Hazard ratio (CI)	*p*
GI infection prior to immunotherapy	1.6 (1.4–1.8)	< 0.001*
Age (increments of 10 years)	1.0 (0.9–1.0)	0.481
Male sex	1.04 (1.01–1.1)	0.023*
White race	0.9 (0.9–1.0)	0.982

*Note:* *Significant at *p* < 0.05.

Abbreviation: GI, gastrointestinal.

### 3.5 Survival Analysis

As seen in Table 5, multivariate Cox proportional hazards survival analysis demonstrated that a GI infection before immunotherapy was associated with worse survival outcomes for patients receiving ICIs than for those without prior GI infections (hazard ratio, 1.6, [95% CI, 1.4–1.8]; *p* < 0.001). Age and race did not significantly impact survival outcomes (*p* > 0.05), and, compared to female sex, male sex was associated with worse survival (hazard ratio, 1.04, [95% CI: 1.01–1.1]; *p* = 0.023).

## 4 Discussion

GI infections disrupt the intestinal microbiome, which can decrease the efficacy of ICI therapy and increase the risk of IRAEs [1, 2]. Previous research has demonstrated that GI infections and antibiotic use during immunotherapy can lead to more severe IMC [3]. To our knowledge, this study is the first to explore the long‐term impact of GI infections before ICI therapy initiation on the risk for GI IRAEs and survival in patients undergoing ICI treatment. We found that, although pre‐ICI therapy infections did not affect IMC severity or outcomes, they did increase the risk of developing colitis and were associated with worse overall survival outcomes.

The GI tract is composed of diverse microflora that play critical roles in physiological functions, including metabolism and inflammation, through the release of short‐chain fatty acids, bile acids, and amino acid‐derived metabolites [16, 17, 18]. These metabolites have implications in antitumor immunity, that is, they can modulate the efficacy of cancer treatments by altering immune composition in the tumor microenvironment [1, 19, 20]. Despite their potential to improve tumor responsiveness to chemotherapy and immunotherapy, specific bacterial strains have been found to predispose patients to develop treatment‐related toxicities, particularly IRAEs. For example, infection with the bacterium *Bacteroides intestinalis* and a decrease in *Firmicutes* bacteria have both been linked to IMC [19, 20, 21]. Conversely, some *Bacteroides* species may confer resistance to colitis [1, 22]. These associations reflect the complexity of the interplay between the intestinal microbiome and underlying metabolite production and immune signaling. For instance, adiponectin is a protein hormone released following the phagocytosis of apoptotic leukocytes that reduces activation of pro‐inflammatory T cells. It has been found to be effective in mitigating IMC symptoms in mouse models [23], and its expression has been suggested to be impacted by alterations in the gut microbiome [24]. Although the clinical significance of these myriad bacterial strains remains uncertain, these findings indicate a clear interaction between the intestinal microbiome and the potency and safety of cancer therapeutics.

Although studies have focused on native gut bacteria, interest in the impact of gut dysregulation on ICI efficacy and toxicity is growing. Prospective human studies on this subject are limited, so proxy measures for dysbiosis, such as antibiotic or proton pump inhibitor use, are often employed. Both antibiotics and proton pump inhibitors have been consistently associated with worse overall survival in patients receiving ICIs, especially when used near the time of ICI therapy initiation. Some studies suggest that antibiotic use near IMC onset increases the risk of severe colitis and that GI infections at the time of IMC diagnosis are an indicator of dysbiosis [6]. Although these findings suggest an acute role for gut dysbiosis in IMC severity, we explored the long‐term effects of baseline gut dysbiosis before ICI therapy. Baseline dysbiosis was approximated by pre‐ICI GI infections and was associated with poor survival and an increased risk of future GI IRAEs. While the precise mechanism of this effect is unknown, there is an increasing volume of research exploring this association in preclinical models with the hopes of clarifying the underlying pathways involved.

GI infections can lead to the long‐term disruption of normal gut flora [2]. For example, examination of postinfection stool samples in one study demonstrated a higher abundance of *Bacteroides* and a decrease of *Faecalibacterium* (in the presence of a controlled infection with *Campylobacter jejuni* ) [25], whereas another study demonstrated reduced taxonomic richness and increased *Proteobacteria*, *Bacteroides*, and *Firmicutes* following diarrheal illness [1, 9, 15]. Typically, the gut microbiome returns to a healthy composition after infection, but the timing is highly variable and can take up to 14 weeks [13, 14, 15].

In our cohort, the median time to colitis onset was about 7 weeks after ICI therapy initiation for patients with GI infections before ICI therapy, suggesting an overlap of postinfectious dysbiosis and IMC development. Additionally, microbiome reconstitution via FMT has been shown to be effective in treating IMC, achieving symptom improvement in up to 85.1% of patients [26]. Similarly, studies by both Elkrief et al. and Halsey et al. observed consistent FMT responses with a symptom resolution rate of 92% after FMT for refractory IMC [27, 28]. Furthermore, a case series by Groenewegen et al. described two patients with treatment‐refractory IMC who were treated with FMT, resulting in decreased stool frequency, tapering of immunosuppressive therapy, hospital discharge, and alterations in gut microbiota composition, richness, and diversity [29]. Notably, in our cohort, patients with prior GI infections were more likely to undergo FMT, plausibly due to the high prevalence of *C. difficile* infection in our cohort. However, we observed no differences in FMT efficacy between the groups, likely owing to our small sample size.

This study had some limitations. First, it was a retrospective study, and all data gathered were limited to those documented in the patients' medical records, which may inaccurately reflect information, and the ability to extract detailed data was limited given our large sample size. Additionally, we relied on automatic data extraction to identify infection status, which restricted our cohort to patients who underwent testing at our institution. Patients who underwent confirmatory stool testing at another institution may have been missed. Although we met the minimum number of patients needed based on our sample size calculation, the power of our study could be further improved by increasing the sample size of patients who developed infection before ICI administration and then had IMC. Finally, the lack of microbiome analysis owing to our study's retrospective design limits our ability to directly validate our findings.

In this study, we explored the impact of GI infection on the efficacy and safety of ICIs. We found that, compared with patients without a history of GI infection, those with such a history were more likely to develop IMC and have worse overall survival outcomes. We suspect this is due to a disruption of the gut microbiome, which has been shown to adversely impact patient survival and increase the risk of treatment‐related toxicities. Prospective studies with stool microbiome analyses are needed to further elucidate the association between gut dysbiosis and ICI‐related toxicities. Nonetheless, baseline gut dysbiosis, as defined by proxies such as GI infections or antibiotic use, may serve as a prognostic marker for ICI treatment efficacy and safety risk.

## Author Contributions

**Malek Shatila:** conceptualization, data curation, writing – original draft. **Kian Abdul‐Baki:** data curation, writing – review and editing. **Andres Urias Rivera:** data curation, writing – review and editing. **Kei Takigawa:** data curation, writing – review and editing. **Irene Jeong‐Ah Lee:** data curation, writing – review and editing. **Andrew Sullivan:** data curation, writing – review and editing. **Tanvi Gupta:** data curation, writing – review and editing. **Linfeng Lu:** data curation, writing – review and editing. **Raakhi Menon:** data curation, writing – review and editing. **Ayesha Khan:** writing – review and editing. **Hamza Salim:** data curation, writing – review and editing. **Elliot Baerman:** data curation, writing – review and editing. **Carolina Colli Cruz:** writing – review and editing, data curation. **Cristina Natha:** writing – review and editing, data curation. **Varun Vemulapalli:** writing – review and editing, data curation. **Garrett Coleman:** data curation, writing – review and editing. **Krishnavathana Varatharajalu:** writing – review and editing, data curation. **Christopher Fan:** writing – review and editing. **Pablo Okhuysen:** writing – review and editing. **Anthony J. Olszanski:** writing – review and editing. **Yan Zhou:** writing – review and editing. **Hao Chi Zhang:** writing – review and editing. **Mehnaz Shafi:** writing – review and editing. **Yinghong Wang:** writing – review and editing, conceptualization.

## Ethics Statement

This study was approved by the MD Anderson Institutional Review Board (PA18‐0472).

## Consent

Patient consent was waived for this study.

## Conflicts of Interest

The authors declare no conflicts of interest.

## Supporting information


**Table S1:** Univariate analysis of the association between gastrointestinal infection prior to ICI use and colitis incidence, as well as the time from ICI therapy to colitis.
**Table S2:** Comparison of colitis outcomes among patients undergoing fecal microbiota transplantation who did or did not have a gastrointestinal infection prior to initiation of immunotherapy (*n* = 58).

## Data Availability

The data that support the findings of this study are available on request from the corresponding author. The data are not publicly available due to privacy or ethical restrictions.
