# Supplementary material for: Gastrointestinal Infection Before Immune Checkpoint Inhibition Hinders Treatment Efficacy and Increases the Risk of Colitis
Source: Cancer Med. 2025 Aug 10;14(15):e71123. doi: 10.1002/cam4.71123 (PMC12336284; doi:10.1002/cam4.71123)
Supplement: Supplementary file 1 — Table S1: Univariate analysis of the association between gastrointestinal infection prior to ICI use and colitis incidence, as well as the time from ICI therapy to colitis. Table S2: Comparison of colitis outcomes among patients undergoing fecal microbiota transplantation who did or did not have a gastrointestinal infection prior to initiation of immunotherapy (n = 58). [file CAM4-14-e71123-s001.docx]

**SUPPLEMENTARY TABLES**

| **Supplementary Table 1. Univariate Analysis of the Association Between Gastrointestinal Infection Prior to ICI Use and Colitis Incidence as well as the Time from ICI Therapy to Colitis** | | |
| --- | --- | --- |
| **Characteristic** | **Odds ratio (CI)** | ***p-*value** |
| Diagnosis of colitis | 1.8 (1.2-2.5) | 0.002* |
| Time from ICI therapy to colitis in months | 0.9 (0.9-1.0) | 0.122 |
| Abbreviation: ICI, immune checkpoint inhibitor.  *Significant at *p* < 0.05. | | |

**Supplementary Table 2.** Comparison of Colitis Outcomes Among Patients Undergoing Fecal Microbiota Transplantation Who Did or Did Not Have a Gastrointestinal Infection Prior to Initiation of Immunotherapy (n = 58)

| **Outcome** | **No. (%)** | | ***p-*value** |
| --- | --- | --- | --- |
|  | **No GI infection prior to ICI (n = 53)** | **GI infection prior to ICI (n = 5)** |  |
| Rehospitalization for IMC | 24 (60.0) | 3 (100) | 0.282 |
| ICI resumption | 17 (33.3) | 3 (60) | 0.336 |
| Symptom resolution | 47 (88.7) | 5 (100) | 1.000 |
| Median duration of symptoms, d (IQR) | 56 (12-112) | 40 (14-70) | 0.924 |
| Endoscopic remission | 27 (79.4) | 2 (50) | 0.233 |

Abbreviations: FMT, fecal microbiota transplantation; GI, gastrointestinal; ICI, immune checkpoint inhibitor; IMC, immune-mediated colitis; IQR, interquartile range.

*Significant at *p* < 0.05.
